# Supplementary material for: An axon-specific expression of HCN channels catalyzes fast action potential signaling in GABAergic interneurons
Source: Nat Commun. 2020 May 7;11:2248. doi: 10.1038/s41467-020-15791-y (PMC7206118; doi:10.1038/s41467-020-15791-y)
Supplement: Supplementary file 1 — Supplementary Information [file 41467_2020_15791_MOESM1_ESM.pdf]

An axon-specific expression of HCN channels catalyzes fast action potential signaling in GABAergic interneurons

Roth and Hu

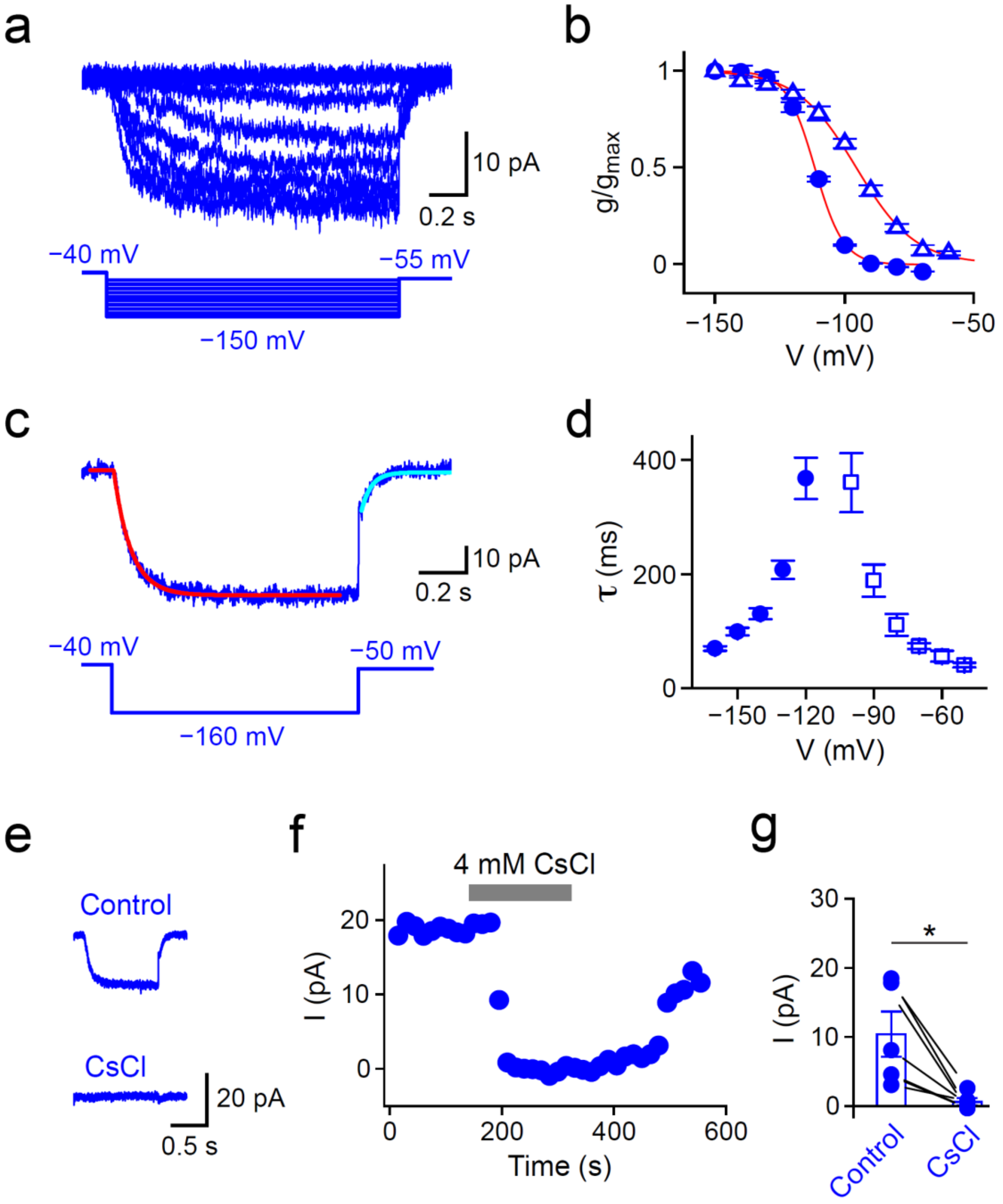

Supplementary Figure 1. Gating and pharmacological properties of h-currents in PV^{+}-BC axons.

(**a**) Top, h-currents in an outside-out patch excised from a PV^{+}-BC axon. Bottom, corresponding voltage pulse protocol with increasing test pulse amplitude.

(**b**) H-conductance activation curve in PV^{+}-BC axons. Blue circles represent data points obtained from axonal outside-out patches (n = 7 axonal outside-out patches); blue triangles represent data points obtained from whole-axon recordings (n = 4 whole-

axon recordings). Red lines represent a Boltzmann function fit to the data points. Consistent with previous work[1,2], the midpoint potential of the h-current activation curve in isolated membrane patches (–111 mV) was more negative than that of whole-axon h-currents (–96 mV). This shift of voltage dependence might be created by differences in Donnan potential between the outside-out patch and whole-cell configuration[3] and the modulation of HCN channel gating by extracellular K^+ ions[4]. Outside-out patches and whole-axon h-current recordings were performed in 120 and 2.5 mM extracellular KCl, respectively.

(**c**) H-current (top) and corresponding voltage pulse (bottom) in an outside-out patch excised from a PV^+-BC axon. Red line represents a monoexponential function with a delay fit to the onset of the current trace to determine the activation time constant, and cyan line represents a monoexponential function fit to the tail current to determine the deactivation time constant.

(**d**) Time constants of h-current activation (filled circles, n = 8 PV^+-BC axonal outside-out patches) and deactivation (open squares, n = 6 PV^+-BC axonal outside-out patches) plotted against voltage.

(**e**) H-currents in an axonal outside-out patch in control (top) and 4 mM extracellular cesium chloride (CsCl, bottom).

(**f**) H-current amplitude plotted against experimental time from the same experiment as in (e).

(**g**) Summary plot of h-current amplitude in control and 4 mM CsCl in 7 axonal outside-out patches. Data from the same experiment are connected by lines. * indicates P = 0.02 (n = 7 PV^+-BC axonal outside-out patches, two-sided Wilcoxon signed rank test).

Error bars in (b), (d) and (g) represent ± SEM.

Source data are provided as a Source data file.

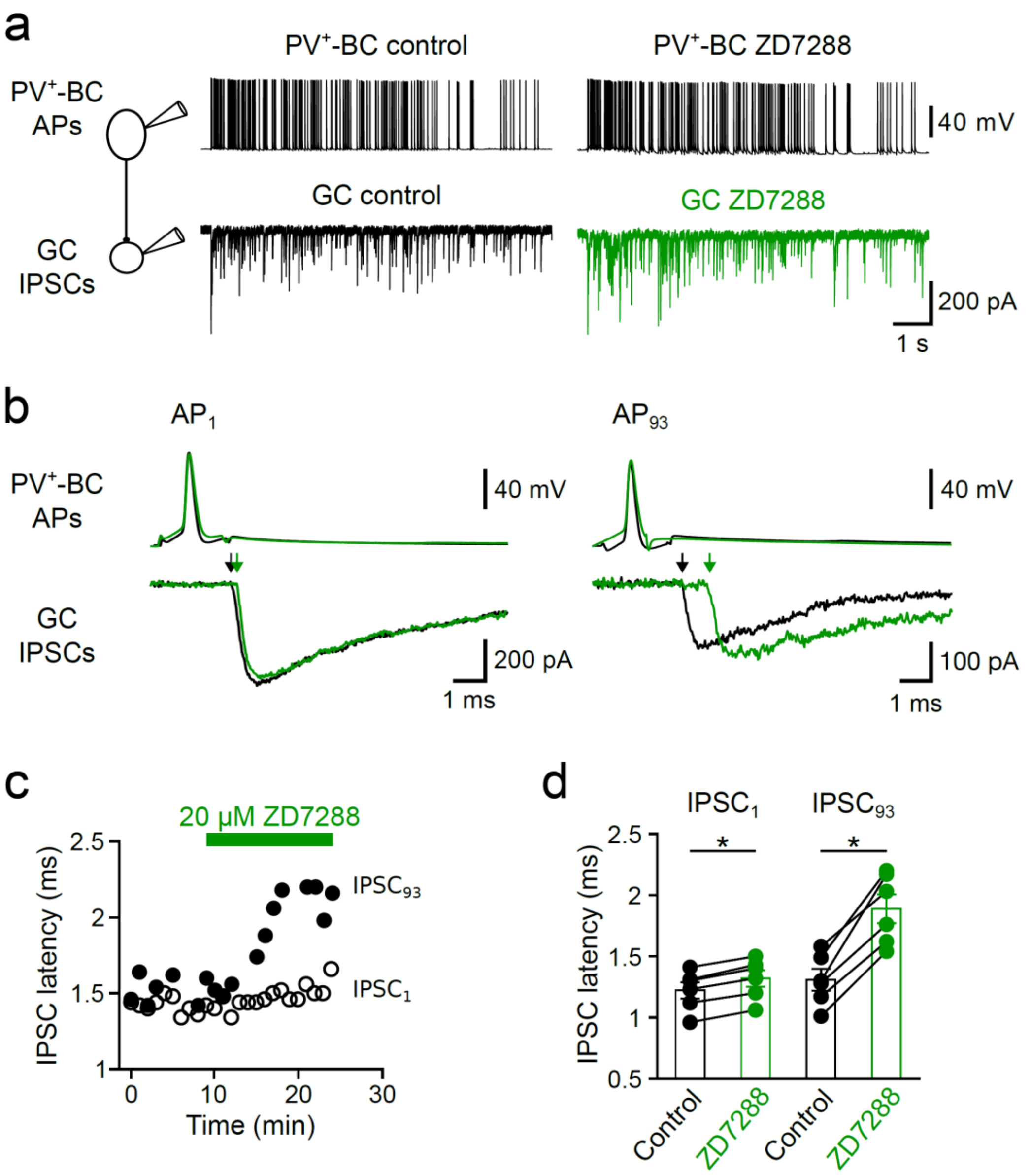

Supplementary Figure 2. HCN channels reduce the latency of unitary ISPCs at PV^{+}-BC–granule cell (GC) synapses.

(**a**) Unitary IPSCs at PV^{+}-BC–GC synapses in the hippocampal dentate gyrus evoked by the physiologically relevant spike pattern before (left) and during the application of 20 µM ZD7288 (right). The physiologically relevant AP pattern in the presynaptic PV^{+}-BC is depicted at the top, and the corresponding IPSC train in the postsynaptic GC is depicted at the bottom. Insert on the left, schematic diagram showing the recording configuration from a monosynaptically connected PV^{+}-BC–GC pair.

(**b**) Left, the first presynaptic AP (AP_1) and corresponding IPSC in control (black traces) and 20 µM ZD7288 (green traces). Right, the 93rd presynaptic AP (AP_{93}) and corresponding IPSC in control (black traces) and 20 µM ZD7288 (green traces). Membrane potentials and currents are aligned using the time point at which presynaptic APs reached the half-maximal peak amplitude in the rising phase as the temporal reference. Arrows indicate the onset of IPSCs.

(**c**) Plot of IPSC latency values against experimental time. Open black circles, latency values of the first IPSC ($IPSC_1$); filled black circles, latency values of the 93rd IPSC ($IPSC_{93}$).

Data in (a)–(c) are from the same experiment.

(**d**) Summary graph of latency values of the first and 93rd IPSC in control and 20 µM ZD7288 in 6 paired PV^+-BC–GC recordings. Data from the same experiment were connected by lines. * indicates P = 0.03 (n = 6 paired PV^+-BC–GC recordings, two-sided Wilcoxon signed rank test). Error bars represent ± SEM.

Source data are provided as a Source data file.

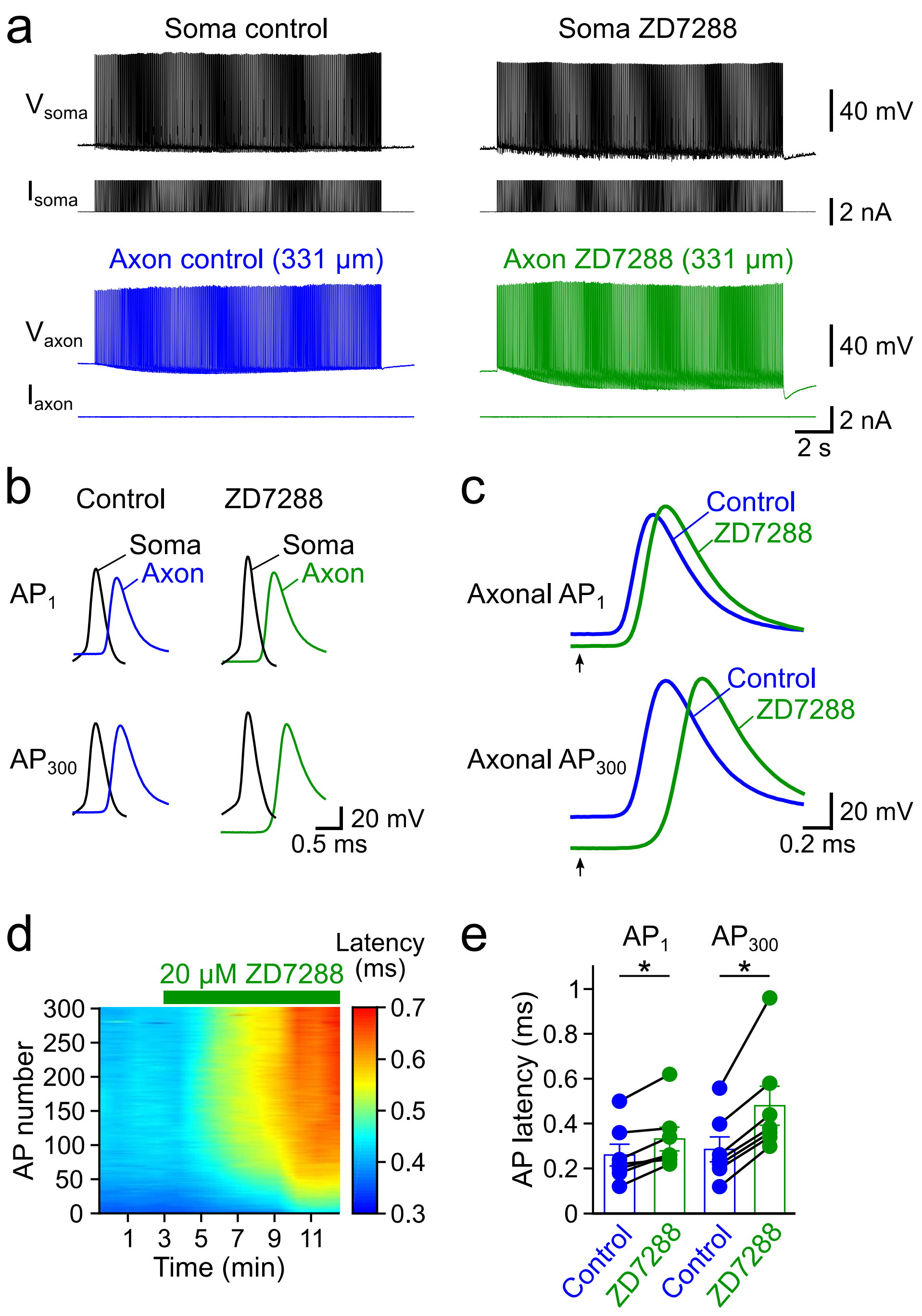

Supplementary Figure 3. HCN channels speed up AP propagation in PV⁺-BC axons during a 20-Hz spike train.

(**a**) Simultaneous soma–axon recording of APs elicited by stimulating the soma at a constant frequency of 20 Hz in control (left) and 20 µM ZD7288 (right).

(**b**) First (top, AP_1) and last (bottom, AP_{300}) APs in the 20-Hz train plotted on an expanded time scale.

(**c**) Superimposition of axonal APs in control and 20 µM ZD7288. Only the first and the last APs in the 20-Hz train are shown. To facilitate the comparison of spike timing, axonal APs were aligned by using the time point (black arrows) at which somatic APs reached the half-maximal peak amplitude in the rising phase as the temporal reference.

Black traces in (a)–(b), somatic voltage and current; blue traces in (a)–(c), axonal voltage and current in control; green traces in (a)–(c), axonal voltage and current in ZD7288.

(**d**) Heat map plotting the latency between every somatic AP and the corresponding axonal spike in the 20-Hz train against experimental time.

Data in (a)–(d) are from the same experiment.

(**e**) Summary graph showing the effect of 20 µM ZD7288 on latencies between somatic and distal axonal APs of the first and 300th spikes in the train. Data from 7 simultaneous soma–axon recordings at distances between 112 to 331 µm. Data points from the same recording are connected by lines. * indicates P = 0.02 (n = 7 soma–axon recordings, two-sided Wilcoxon signed rank test). Error bars represent ± SEM.

Source data are provided as a Source data file.

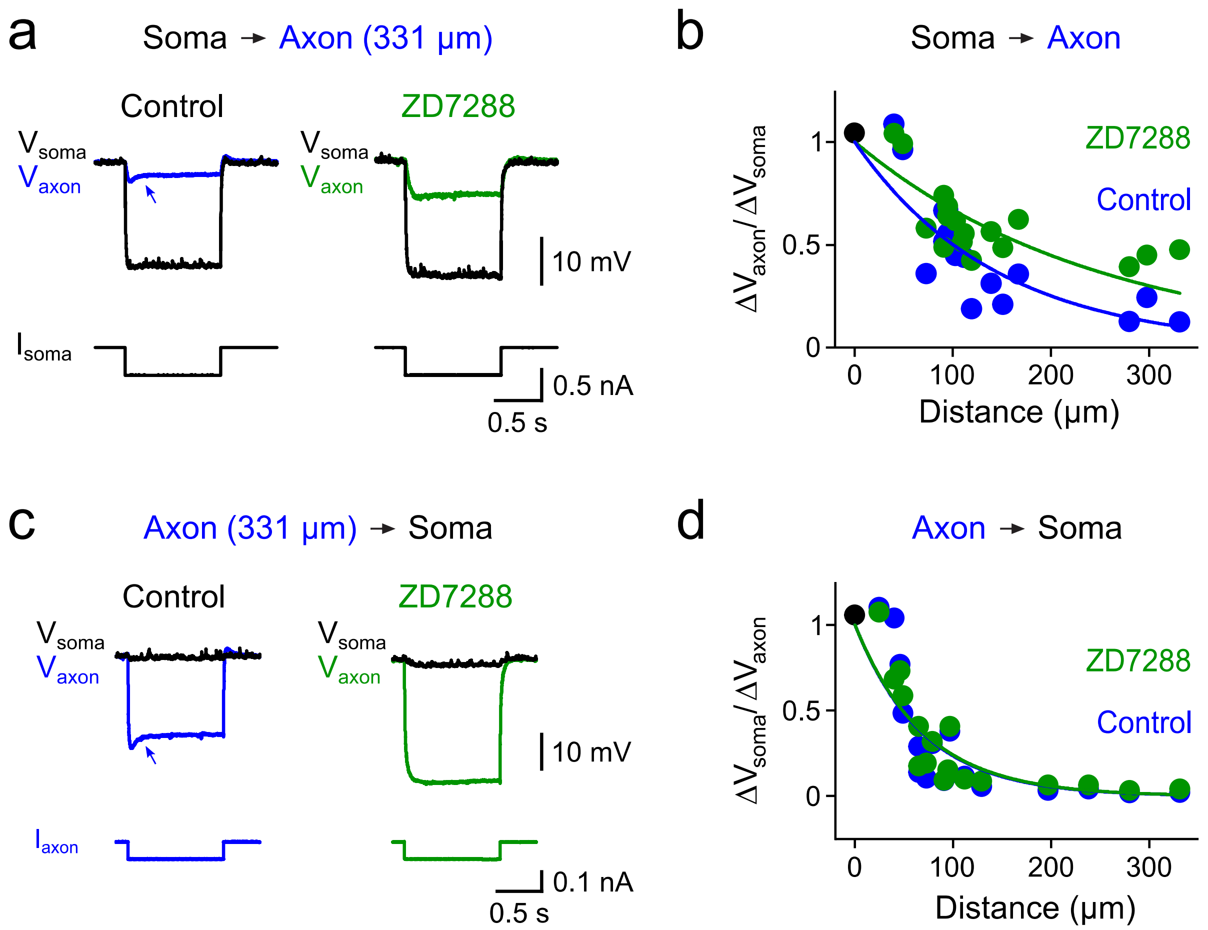

Supplementary Figure 4. Blocking HCN channels enhanced steady-state voltage transfer from PV$^+$-BC somata to axons.

(**a**) Superimposition of somatic and axonal voltage responses to the somatic injection of a 1-s hyperpolarizing current pulse to determine the voltage transfer in the soma-to-axon direction in control (left) and 20 μM ZD7288 (right). Black traces, somatic voltage and current; blue traces, axonal voltage in control; green traces, axonal voltage in ZD7288. Blue arrow indicates the depolarizing 'sag' in the axonal voltage response in control. Note that the 'sag' was inhibited by ZD7288.

(**b**) Summary plot showing the transfer of steady-state voltage signals from the soma to the axon in PV$^+$-BCs as a function of distance between somatic and axonal recording sites before and during the application of ZD7288. Voltage transfer was quantified by dividing the steady-state amplitude of the voltage response in the axon by that of the corresponding somatic voltage response ($\Delta V_{axon}/\Delta V_{soma}$). Blue circles, data points in control; green circles, data points in ZD7288. Lines represent a monoexponential function to determine the axonal length constant in the soma-to-axon direction.

(**c**) From the same experiment in (a), but the 1-s hyperpolarizing current pulse was injected into the axon to determine voltage transfer from the axon to the soma.

(**d**) Similar as in (b), but showing the transfer of steady-state voltage signals from the axon to the soma.

Data in (b) and (d) are from one somatic recording and 17 soma–axon recordings at distances between 24 to 331 µm. HCN channels were blocked with 10 µM ZD7288 in 2 recordings and with 20 µM ZD7288 in the remaining 16 recordings.

Source data are provided as a Source data file.

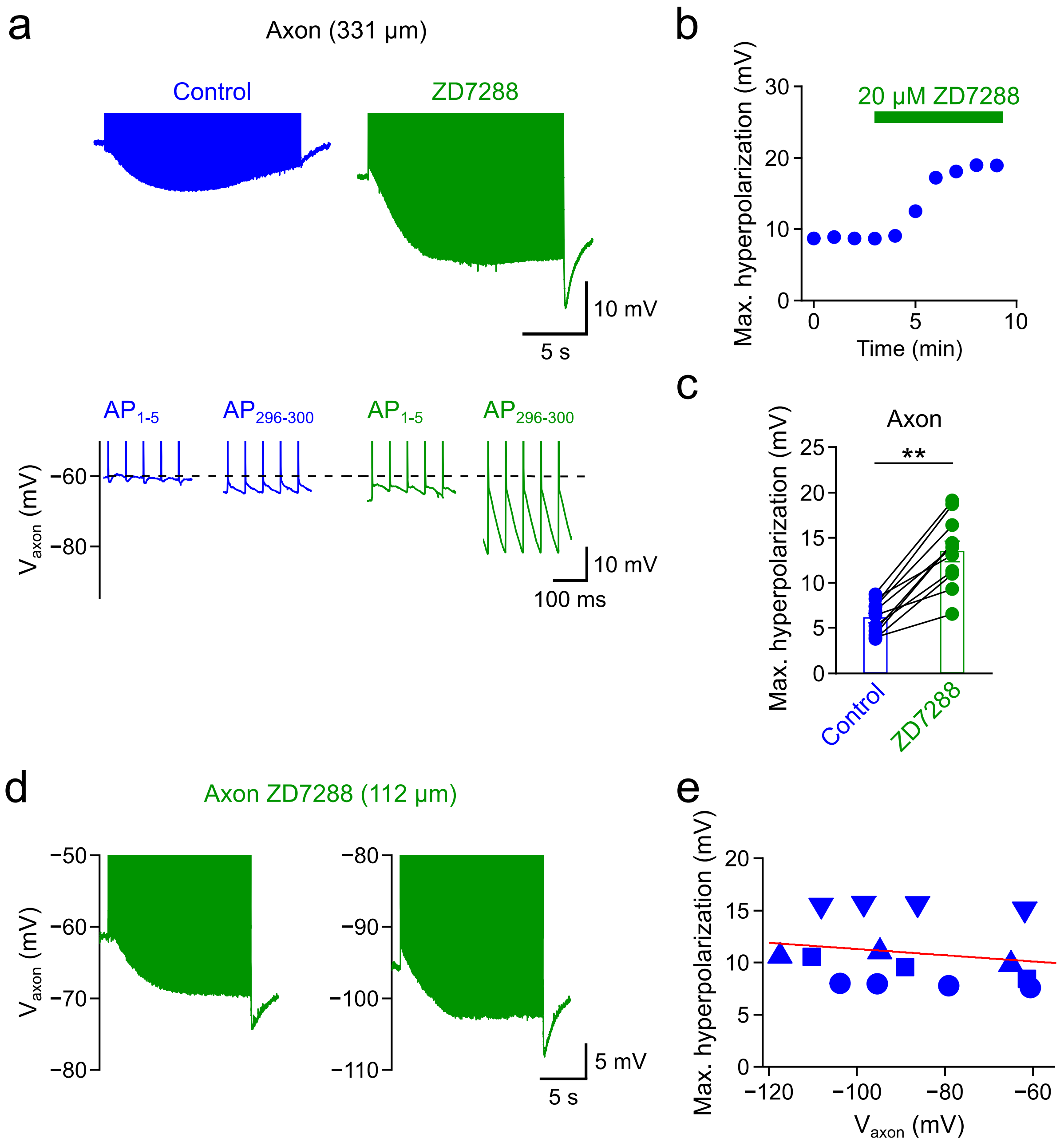

Supplementary Figure 5. ZD7288 unmasked an AP-dependent hyperpolarization in PV^{+}-BC axons during the 20-Hz AP train.

(**a**) Top, axonal membrane potential trajectories in control (blue) and 20 μM ZD7288 (green). Bottom, axonal voltage between the first and fifth APs ($AP_{1–5}$) as well as that between the 296th and 300th APs ($AP_{296–300}$) plotted on an expanded time scale. APs were truncated at –55 mV (top) and –50 mV (bottom) to highlight the hyperpolarization between APs.

(**b**) Peak amplitude of the AP-dependent hyperpolarization (Max. hyperpolarization) in the axon plotted against experimental time from the experiment in (a).

(**c**) Summary graph comparing the peak amplitude of the AP-dependent hyperpolarization during the 20-Hz train in 11 PV^+-BC axons under control conditions (blue circles) with that in 20 μM ZD7288 (green circles). Data points from the same recording are connected by lines. ** indicates P = 0.001 (n = 11 soma–axon recordings, two-sided Wilcoxon signed rank test). Error bars represent ± SEM.

(**d**) Comparison of the AP-dependent hyperpolarization in a PV^+-BC axon at two different baseline membrane potentials. A constant current was injected into the axon to adjust the baseline membrane potential (left, –61 mV; right, –95 mV). 20 μM ZD7288 was continuously present in the bath solution during this recording.

(**e**) The amplitude of the AP-dependent axonal hyperpolarization in 20 μM ZD7288 plotted against the baseline axonal membrane potential in 4 separate recordings. Data from different recordings were represented by different symbols. Red line represents a linear function fit to the data points (Spearman ρ = –0.35, P = 0.22, n = 4 soma–axon recordings).

Source data are provided as a Source data file.

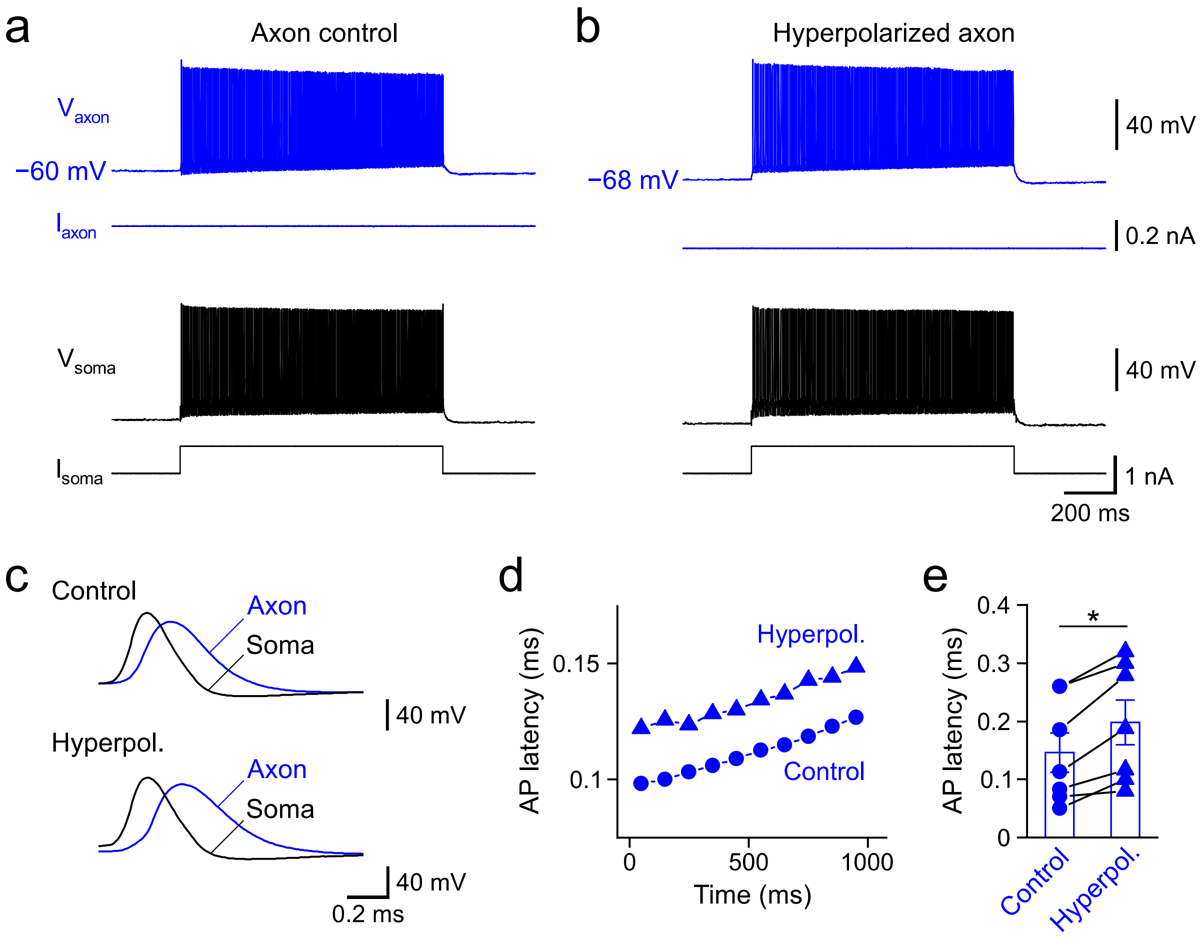

Supplementary Figure 6. Hyperpolarizing PV$^+$-BC axons by constant current injection increased the latency between somatic and axonal APs.

(**a**) A train of APs evoked by injecting a 1-s depolarizing current pulse into the soma during a simultaneous soma–axon recording of a PV$^+$-BC under control conditions at the RMP (–60 mV). The distance between somatic and axonal patch sites was 123 µm.

(**b**) Same as in (a), except that the axonal baseline membrane potential was hyperpolarized to –68 mV by injecting a constant current (–150 pA) via the axonal recording pipette.

(**c**) Top, superimposition of the first somatic AP in the train shown in (a) over the corresponding axonal AP. Bottom, superimposition of the first somatic AP in the train shown in (b) over the corresponding axonal AP. Note that hyperpolarizing the axon to –68 mV by injecting a constant current increased the latency between the somatic and axonal APs.

(**d**) The latency between somatic and axonal APs plotted against the time from the onset of the 1-s somatic current pulse that evoked the AP train. The 1-s AP train was divided into 10 equally spaced time bins, and each data point represents the mean of soma–axon AP latencies of all spikes in a single bin. Blue circles, latency values from the traces in (a), where the axonal baseline membrane potential was at –60 mV under control conditions; blue triangles, latency values from the traces in (b), where the axonal baseline membrane potential was hyperpolarized to –68 mV by constant current injection.

Data in (a)–(d) are from the same recording.

(**e**) Summary graph of 7 simultaneous soma–axon recordings showing that hyperpolarizing the axonal membrane potential from –61.3 ± 0.9 mV (Control, filled circles) to –74.8 ± 1.8 mV (Hyperpolarized, filled triangles) by injecting constant currents into the axon increased the latency between somatic and corresponding axonal APs. Data from the same experiment are connected by lines. * indicates P = 0.02 (n = 7 soma–axon recordings, two-sided Wilcoxon signed rank test). Error bars represent ± SEM.

Source data are provided as a Source data file.

Supplementary Table 1. RMP, input resistance and axonal length constant values in control and ZD7288.

		**Control**	**ZD7288**	**P value**
RMP	Soma (n = 22)	−61.9 ± 0.8 mV	−64.6 ± 0.9 mV	4.3×10^{-5}
	Axon (n = 13)	−58.1 ± 0.9 mV	−61.0 ± 0.9 mV	0.002
Input resistance	Soma (n = 25)	58.1 ± 3.7 MΩ	71.0 ± 4.3 MΩ	1.3×10^{-5}
	Axon (n = 20)	268.9 ± 50.3 MΩ	361.8 ± 62.9 MΩ	0.0001
Axonal length constant	From soma to axon (n = 18)	144.7 µm	249.4 µm	0.002
	From axon to soma (n = 18)	69.0 µm	70.1 µm	0.311

P values in the comparisons of RMP and input resistance were determined with two-sided Wilcoxon signed rank tests, whereas P values in the comparisons of axonal length constant were determined with one-way analyses of covariance (ANCOVA). No adjustment was made for multiple comparisons.

Source data are provided as a Source data file.

Supplementary references

1. Angelo, K., London, M., Christensen, S. R. & Häusser, M. Local and global effects of I_h distribution in dendrites of mammalian neurons. *J. Neurosci.* **27,** 8643–8653 (2007).
2. Williams, S. R., Christensen, S. R., Stuart, G. J. & Häusser, M. Membrane potential bistability is controlled by the hyperpolarization-activated current I_H in rat cerebellar Purkinje neurons *in vitro*. *J. Physiol.* **539,** 469–483 (2002).
3. Fenwick, E. M., Marty, A. & Neher, E. Sodium and calcium channels in bovine chromaffin cells. *J. Physiol.* **331,** 599–635 (1982).
4. Azene, E. M., Xue, T. & Li, R. A. Molecular basis of the effect of potassium on heterologously expressed pacemaker (HCN) channels. *J. Physiol.* **547,** 349–356 (2003).
