# Supplementary material for: An axon-specific expression of HCN channels catalyzes fast action potential signaling in GABAergic interneurons
Source: Nat Commun. 2020 May 7;11:2248. doi: 10.1038/s41467-020-15791-y (PMC7206118; doi:10.1038/s41467-020-15791-y)
Supplement: Supplementary file 3 — Reporting Summary [file 41467_2020_15791_MOESM3_ESM.pdf]

nature research

Corresponding author(s): Hua Hu

Last updated by author(s): Mar 20, 2020

Reporting Summary

Nature Research wishes to improve the reproducibility of the work that we publish. This form provides structure for consistency and transparency in reporting. For further information on Nature Research policies, see Authors & Referees and the Editorial Policy Checklist.

Statistics

For all statistical analyses, confirm that the following items are present in the figure legend, table legend, main text, or Methods section.

n/a	Confirmed	
☐	☒	The exact sample size (*n*) for each experimental group/condition, given as a discrete number and unit of measurement
☐	☒	A statement on whether measurements were taken from distinct samples or whether the same sample was measured repeatedly
☐	☒	The statistical test(s) used AND whether they are one- or two-sided<br>*Only common tests should be described solely by name; describe more complex techniques in the Methods section.*
☒	☐	A description of all covariates tested
☒	☐	A description of any assumptions or corrections, such as tests of normality and adjustment for multiple comparisons
☐	☒	A full description of the statistical parameters including central tendency (e.g. means) or other basic estimates (e.g. regression coefficient) AND variation (e.g. standard deviation) or associated estimates of uncertainty (e.g. confidence intervals)
☐	☒	For null hypothesis testing, the test statistic (e.g. *F*, *t*, *r*) with confidence intervals, effect sizes, degrees of freedom and *P* value noted<br>*Give P values as exact values whenever suitable.*
☒	☐	For Bayesian analysis, information on the choice of priors and Markov chain Monte Carlo settings
☒	☐	For hierarchical and complex designs, identification of the appropriate level for tests and full reporting of outcomes
☒	☐	Estimates of effect sizes (e.g. Cohen's *d*, Pearson's *r*), indicating how they were calculated

Our web collection on statistics for biologists contains articles on many of the points above.

Software and code

Policy information about availability of computer code

Data collection	pClamp V9.2.1.9 & V10.3.1.4 (Molecular Devices)
Data analysis	Electrophysiological recordings were analyzed using Stimifit (https://github.com/neurodroid/stimfit), Clampfit V9 & V10 (Molecular Devices), Origin 2015-2017 (OriginLab), Matlab 2018a (Mathworks), Excel 2016 (Microsoft) and Minitab 17 (Minitab).

For manuscripts utilizing custom algorithms or software that are central to the research but not yet described in published literature, software must be made available to editors/reviewers. We strongly encourage code deposition in a community repository (e.g. GitHub). See the Nature Research guidelines for submitting code & software for further information.

Data

Policy information about availability of data

All manuscripts must include a data availability statement. This statement should provide the following information, where applicable:

- Accession codes, unique identifiers, or web links for publicly available datasets
- A list of figures that have associated raw data
- A description of any restrictions on data availability

The data underlying the findings of this study are available from the corresponding author upon reasonable request. The source data underlying Figs 1c–d, 2e, i, 3e–h, 4d, 5c, e, g, Supplementary Figs 1b, d, g, 2d, 3e, 4b, d, 5c, 6e and Supplementary Table 1 are provided as a Source Data file.

Field-specific reporting

Please select the one below that is the best fit for your research. If you are not sure, read the appropriate sections before making your selection.

☒ Life sciences ☐ Behavioural & social sciences ☐ Ecological, evolutionary & environmental sciences

For a reference copy of the document with all sections, see nature.com/documents/nr-reporting-summary-flat.pdf

Life sciences study design

All studies must disclose on these points even when the disclosure is negative.

Sample size	Because of the difficulty to obtain subcellular patch-clamp recordings from interneuron axons and dendrites, the sample size was limited by the number of experiments. However, we determined the sample size in this paper based on the published numbers in the field. Because the number of recordings in each figure is similar or exceeds those in previous publications (for example, Hu H and Jonas P, 2014, Nature Neuroscience), the sample size is sufficient to detect differences between conditions.
Data exclusions	Data points were excluded if the resting membrane potential was more positive than -50 mV, which indicates that the cell was unhealthy and unsuitable for the analyses.<br>Furthermore, the distance between somatic and axonal recording sites could not be determined in 8 experiments, in which the axonal recording site could not be traced back to the soma. Without distance values, these data points are excluded from the analysis of distance-dependence.<br>Finally, experiments in which the axon originated from one of the dendrites were excluded from the analyses to determine AP propagation speed and axonal voltage transfer, because of contamination created by AP propagation delay and steady-state voltage attenuation in dendrites.<br>The exclusion criteria were pre-established.
Replication	All experiments have been successfully repeated with different batches of animals over extended periods of time. A subset of the experiments were repeated by different researchers. All replications were successful.
Randomization	No randomization was used in this study. When comparing the response of a cell in control with that of the same cell in ZD7288 in pharmacological experiments, randomization was not possible because the effect of ZD7288 is nearly irreversible.
Blinding	No blinding was used in this study because the experimenter had to apply pharmacological channel blockers himself during patch-clamp recordings. In a subset of experiments, however, patch-clamp recording and the analysis of the recorded data were performed independently by two separate researchers.

Reporting for specific materials, systems and methods

We require information from authors about some types of materials, experimental systems and methods used in many studies. Here, indicate whether each material, system or method listed is relevant to your study. If you are not sure if a list item applies to your research, read the appropriate section before selecting a response.

Materials & experimental systems

n/a	Involved in the study	
☒	☐	Antibodies
☒	☐	Eukaryotic cell lines
☒	☐	Palaeontology
☐	☒	Animals and other organisms
☒	☐	Human research participants
☒	☐	Clinical data

Methods

n/a	Involved in the study	
☒	☐	ChIP-seq
☒	☐	Flow cytometry
☒	☐	MRI-based neuroimaging

Animals and other organisms

Policy information about studies involving animals; ARRIVE guidelines recommended for reporting animal research

Laboratory animals	Wistar rats (P17 to P23), male, Strain code 273, Charles River laboratories, Sulzfeld, Germany
Wild animals	No wild animals were used in this study.
Field-collected samples	No field-collected samples were used in this study.
Ethics oversight	Experiments on Wistar rats were ethically approved by the Norwegian Food Safety Authority (Mattilsynet).

Note that full information on the approval of the study protocol must also be provided in the manuscript.
